# Navigation-Assisted One-Staged Posterior Spinal Fusion Using Pedicle Screw Instrumentation in Adolescent Idiopathic Scoliosis—A Case Series

**DOI:** 10.3390/medicina60020300

**Published:** 2024-02-09

**Authors:** Pao-Lung Chang, Michael Jian-Wen Chen, Pang-Hsuan Hsiao, Chia-Yu Lin, Yuan-Shun Lo, Chun Tseng, Ling-Yi Li, Chien-Ying Lai, Hsien-Te Chen

**Affiliations:** 1 Department of Education, China Medical University Hospital, China Medical University, Taichung 404, Taiwan; paul880727@gmail.com; 2 Department of Orthopaedic Surgery, China Medical University Hospital, China Medical University, Taichung 404, Taiwan; mchen115@gmail.com (M.J.-W.C.); rayxhiao@gmail.com (P.-H.H.); chiayulin1213@gmail.com (C.-Y.L.); yuanshunlo@gmail.com (Y.-S.L.); orthodrtseng@gmail.com (C.T.); cloud8935@gmail.com (L.-Y.L.); 017110@tool.caaumed.org.tw (C.-Y.L.); 3 Spine Center, China Medical University Hospital, China Medical University, Taichung 404, Taiwan; 4 Department of Orthopedic Surgery, China Medical University Beigang Hospital, China Medical University, Yunlin County 651, Taiwan; 5 Graduate Institute of Precision Engineering, National Chung Hsing University, Taichung 402, Taiwan; 6 Graduate Institute of Biomedical Sciences, China Medical University, Taichung 404, Taiwan; 7 Department of Sport Medicine, College of Health Care, China Medical University, Taichung 404, Taiwan

**Keywords:** adolescent idiopathic scoliosis, navigation, posterior fusion, scoliosis surgery, surgical complications

## Abstract

*Background and Objectives*: Adolescent idiopathic scoliosis (AIS) is a prevalent three-dimensional spinal disorder, with a multifactorial pathogenesis, including genetics and environmental aspects. Treatment options include non-surgical and surgical treatment. Surgical interventions demonstrate positive outcomes in terms of deformity correction, pain relief, and improvements of the cardiac and pulmonary function. Surgical complications, including excessive blood loss and neurologic deficits, are reported in 2.27–12% of cases. Navigation-assisted techniques, such as the O-arm system, have been a recent focus with enhanced precision. This study aims to evaluate the results and complications of one-stage posterior instrumentation fusion in AIS patients assisted by O-arm navigation. *Materials and Methods*: This retrospective study assesses 55 patients with AIS (12–28 years) who underwent one-stage posterior instrumentation correction supported by O-arm navigation from June 2016 to August 2023. We examined radiological surgical outcomes (initial correction rate, loss of correction rate, last follow-up correction rate) and complications as major outcomes. The characteristics of the patients, intraoperative blood loss, operation time, number of fusion levels, and screw density were documented. *Results*: Of 73 patients, 55 met the inclusion criteria. The average age was 16.67 years, with a predominance of females (78.2%). The surgical outcomes demonstrated substantial initial correction (58.88%) and sustained positive radiological impact at the last follow-up (56.56%). Perioperative complications, including major and minor, occurred in 18.18% of the cases. Two patients experienced a major complication. Blood loss (509.46 mL) and operation time (402.13 min) were comparable to the literature ranges. Trend analysis indicated improvements in operation time and blood loss over the study period. *Conclusions*: O-arm navigation-assisted one-stage posterior instrumentation proves reliable for AIS corrective surgery, achieving significant and sustained positive radiological outcomes, lower correction loss, reduced intraoperative blood loss, and absence of implant-related complications. Despite the challenges, our study demonstrates the efficacy and maturation of this surgical approach.

## 1. Introduction

Adolescent idiopathic scoliosis (AIS) is a complex three-dimensional structural spinal disorder that exerts a substantial impact on the physical and mental health of an individual. According to the current literature, the prevalence of AIS is relatively high, with an estimated global prevalence of 0.47–5.2% [1]. Gender is a significant factor in the epidemiology of AIS; the ratio of females to males varies from 1.5:1 to 3:1 and increases significantly with age [2,3]. Sung et al. reported the highest incidence of AIS in Korean females aged 10 to 14, with urban areas showing higher rates than rural areas [4]. A French study linked AIS with obesity, revealing a higher prevalence in individuals with obesity than in the general population [5]. The pathogenesis of AIS is multifactorial and unclear, lacking a single causative factor. Alla et al. indicated that while AIS typically manifests around puberty, it has epigenetic onset during early embryogenesis [6,7]. Gisselle et al. revealed that several genes are associated with the Wnt/β–catenin signaling pathways, which is a hot-spot pathway in AIS pathogenesis [8]. According to the current literature, the pathogenesis of AIS has often been associated with several specific aspects, including bone marrow mesenchymal stem cells, genetic analysis, tissue analysis, spine biomechanics, neurologic analysis, endocrines, biochemical analysis, environmental factors, and lifestyle exploration [9].

Observation, rehabilitation, bracing, and surgery are treatment options for AIS. The prevailing consensus suggests that surgery should be considered for Cobb angles measuring over 40 to 45 degrees, particularly when the patient has growth potential [10,11]. Instrumentation-based spinal surgery can effectively rectify a substantial part of the deformity and aims to prevent further progression of the scoliotic curve. Previous studies reported that the mean major Cobb angle correction rate after AIS corrective surgery ranged from 58.7% to 71.4% [12,13,14]. Meanwhile, surgical treatment can not only provide an improvement in radiological outcomes, but is also significantly positively correlated with pain, quality of life, self-image, and satisfaction after surgery [15,16]. Severe scoliosis might even impact cardiac and lung function. Sarwahi et al. reported that increased tricuspid regurgitant jet velocity and right ventricular systolic pressure were associated with patients with AIS. Postsurgery, these abnormal values normalized, indicating a positive impact on cardiac function [17]. A study reported that residual capacity volume and the total lung capacity increased significantly after surgery, indicating the potential pulmonary benefits of scoliosis surgery [18].

Despite the advantage of surgery, the perioperative complications of corrective surgery for AIS were reported in various ways from 2.27% to 12% [19]. Another study has reported that the complication rate of AIS surgery between 2010 and 2013 improved to 5.09% compared to surgery between 1995 and 1999, with a complication rate of 18.7% [20]. Among surgical complications of AIS, excessive blood loss has been one of the most common complications [21]. Excessive intraoperative blood loss was significantly associated with several factors, including the gender of the patient and the long duration of surgery [22]. Moreover, Xuerong et al. reported an increased risk of blood loss of more than 30% of the estimated total blood volume while the patient had a preoperative Cobb angle larger than 50 degrees or planned to receive fusion of more than six levels [23]. Neurological deficits can be serious iatrogenic complications that occur in scoliosis surgery, including both sensory and motor deficits. The rate of neurologic complication of scoliosis surgery ranged from 0.7% to 2.0% [24]. Therefore, the navigation system for scoliosis surgery, aimed at decreasing the rate of implant-related complications, has been a focus in recent years.

The navigation-assisted technique in spine surgery has been increasingly utilized year by year in the past decade. A previous study reported that the use of O-arm-based techniques significantly improved the precision and safety of pedicle screw insertion, providing a viable alternative to the freehand technique in scoliosis surgery [25]. The O-arm navigation system enables precise intraoperative CT imaging for accurate surgical instrumentation but raises concerns due to increased higher radiation exposure compared to X-ray radiography, especially in pediatric surgery. However, Kazuyoshi et al. reported a total radiation exposure utilizing an O-arm navigation system roughly equivalent to preoperative CT scans [26]. Furthermore, O-arm navigation for pedicle screw instrumentation did not prolong the operating time, was not associated with increased blood loss, and lowered the risk of screw misplacement compared to freehand and fluoroscopy-guided instrumentation [27].

In the past decade, there has been a greater focus on navigation-assisted posterior instrumentation [28,29]. However, few studies have focused on the outcome and complication of navigation-assisted one-stage posterior instrumentation in AIS [30]. As a result, the objective of this study was to evaluate outcomes and complications after navigation-assisted one-stage posterior instrumentation for corrective surgery for AIS.

## 2. Materials and Methods

### 2.1. Study Design and Setting

This is a retrospective case series of collected data from AIS patients who received O-arm navigation-assisted one-stage posterior instrumentation correction at China Medical University Hospital from June 2016 to August 2023. This study was approved by the Research Ethics Committee of China Medical University Hospital (approval number: CMUH109-REC1-009) and conducted in accordance with the ethical principles of the Declaration of Helsinki.

### 2.2. Participants

The inclusion criteria enrolled all AIS patients (across all Lenke types) who underwent navigation-assisted one-stage posterior instrumentation correction surgery within the age range of 12 to 28 years. The diagnosis of adolescent idiopathic scoliosis was established for those patients operated on under the age of 18 years. The diagnosis of AIS was made clinically and radiographically. Diagnostic criteria include age ≥ 10 years, deviation of the spine in the coronal plane characterized by a Cobb angle exceeding 10 degrees with associated clinical rotation of the spinal column, and absence of other etiologies. We excluded patients with syndromic scoliosis, congenital scoliosis, patients with incomplete data, and those who underwent revision surgery or anterior approach surgery. If the patient underwent revision surgery, we only included data of the initial surgery and follow-up data prior to the revision surgery. A total of 73 patients underwent O-arm navigation-assisted one-stage posterior instrumentation correction surgery from June 2016 to August 2023. Of those, 55 patients met the inclusion criteria, while 18 patients were excluded. Among the 55 patients that met the inclusion criteria, none of the patients dropped out from our periodic follow ups.

### 2.3. Variables

Data on patient characteristics, including age, sex, height, body weight, and body mass index, were collected during admission to surgery. Preoperative, intraoperative, and perioperative data, including the number of instrumentation levels, number of instrumented screws, screw density, Cobb angle, operation time, intraoperative blood loss, preoperative and postoperative hemoglobin (Hb) levels, allogeneic blood transfusion utilization, length of hospitalization, Lenke type and the complication rate, were systematically documented.

### 2.4. Surgical Procedure and Postoperative Care

**Figure 1:**
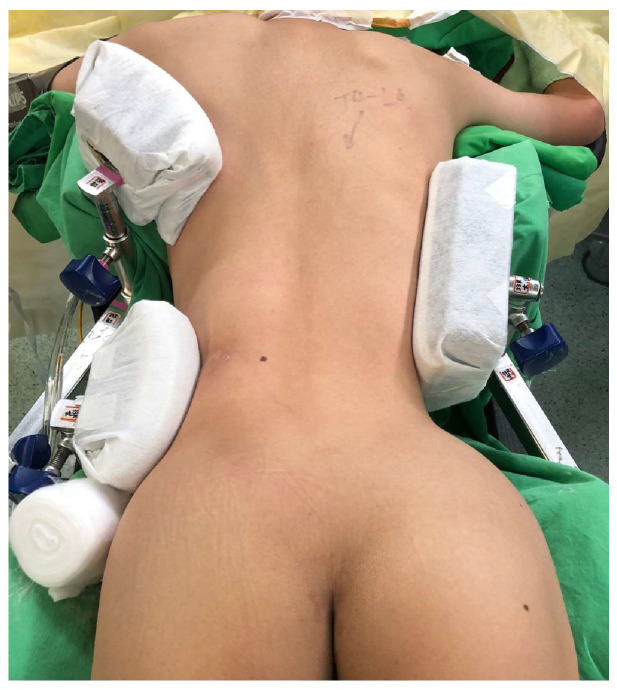
The compression stabilization maneuver before operation, a major compression force at the scoliotic apex with a counteraction force on the opposite cranial and caudal trunk to achieve partial correction of the scoliotic curve, stabilizes the patient on the surgical table.

After inducing general anesthesia and setting intraoperative neuromonitoring (Orthopedic Systems Inc., Union City, CA, USA), lateral support pads are mounted on the side rail, allowing a strong compression force at the apex of the spinal curvature with a counteraction force on the opposite cranial and caudal trunk to achieve partial correction of the scoliotic curve and stabilize the patient on the surgical table (Figure 1). This compression stabilization maneuver before operation is a key step in reducing the inaccuracy of navigation. Following thorough padding and patient positioning on the surgical table, the O-arm image system is activated to assess the machine’s movement path, preventing any collisions with surgical table accessories during navigation. The surgical team drapes the surgical field in a sterile fashion and marks the planned incision sites. The surgeon begins with a midline incision over the affected spinal segments, followed by careful dissection through the muscle layers to expose the base of the transverse process and facets for insertion of the pedicle. Subperiosteal dissection exposes the bony elements and preserves the spinal process while minimizing blood loss and muscle trauma. Using O-arm navigation guidance, pedicle screws are meticulously inserted into the vertebral bodies at predetermined levels on both sides of the spine. Global rod derotation, vertebral translation, and segmental compression/distraction techniques are implemented for scoliosis correction. Synthetic bone graft material is packed onto the surfaces of the decorticated lamina bone to promote fusion. Once alignment correction and bone grafting are completed, the surgeon confirms optimal positioning through intraoperative imaging. Wound closure involves layer-by-layer reapproximation of soft tissues and skin closure with sutures.

Patients gradually transition from bed rest to mobilization under the guidance of physical therapy. Every patient wore a thoracolumbar spinal brace for 2–3 months, and pain medication was discontinued 1 month after the operation. Periodic follow-ups 1, 3, 6, 12 months, and annually and imaging follow the progress of spinal fusion and overall recovery.

### 2.5. Measurements

The evaluation of surgical outcome includes the satisfactory rate of operation, initial correction rate, loss of correction rate, and last follow-up correction rate. The initial correction rate was measured by the change between the preoperative major Cobb angle and the postoperative initial major Cobb angle divided by the preoperative major Cobb angle. Loss of correction rate was defined as the change between the postoperative initial major Cobb angle and the major Cobb angle measured during the last follow-up divided by the initial correction angle. The last follow-up correction rate was measured by the difference between the preoperative major Cobb angle and the major Cobb angle measured during the last follow-up divided by the preoperative major Cobb angle.

We systematically examined and classified all major and minor complications according to the severity criteria proposed by the Harms Study Group [31]. Major complications were identified if they required reoperation, posed a life-threatening risk, or resulted in injury to the spinal cord or nerve root. Any initially classified minor complication could be upgraded to major if it led to prolonged hospitalization of more than two days, required readmission, or required revision surgery. We define complications that occur within 12 weeks after surgery as ‘perioperative’.

During surgery, an intraoperative blood salvage system was utilized. Intraoperative total blood loss was estimated by subtracting the total fluid used for intraoperative irrigation from the final volume accumulated in the reservoir. Major blood loss was defined as blood loss greater than the estimated body volume, calculated according to Nadler’s formula (female adolescent: 65 mL/kg, male adolescent: 70 mL/kg).

### 2.6. Statistical Analysis

Categorical variables were represented by numbers (n) and percentages (%), while continuous variables were described as mean ± standard deviation (SD). Subgroup analysis utilized an independent sample *t*-test to examine potential significant differences in perioperative outcome between two age groups. All reported *p*-values were two-tailed, with a significance level set at 0.05. All differences were considered significant at *p* < 0.05. All statistical analyses were performed with SPSS (version 29; IBM Corporation, Armonk, NY, USA).

## 3. Results

Among 55 patients that met the inclusion criteria, 32 patients (58.2%) received surgery in their early adolescent stage (age ≤ 16) and 22 (41.8%) received surgery in their late adolescent stage or adulthood. Among the 55 included patients, 43 were female (78.2%). The average age of the patients was 16.67 ± 3.32 years, and their mean height and weight were 157.09 ± 8.49 cm and 48.49 ± 9.11 kg, respectively. The body mass index (BMI) of the patients was mostly within normal range, with an average BMI of 19.66 ± 3.49. The mean hemoglobin level prior to surgery was 13.50 ± 1.42 g/dL and the mean preoperative major Cobb angle was 58.24 ± 15.99 degree. The distribution by Lenke type revealed that 23.6% were classified as type 1, 34.5% as type 2, 10.9% as type 3, 5.5% as type 4, 9.1% as type 5, and 9.1% as type 6. The mean follow-up time was 932.65 days.

The operative data are presented in terms of the mean ± standard deviation values, except in the case of allogenic blood transfusion. Notable findings include an average hospitalization period of 6.62 ± 1.16 days and a mean duration of surgery of 402.13 ± 117.57 min. The operative data revealed a mean utilization of 18.87 ± 3.46 screws per case, an average of 11.55 ± 1.92 level instrumented and a screw density of 1.65 ± 0.25 per level. Following surgery, postoperative hemoglobin levels averaged 11.10 ± 1.57 g/dL, and intraoperative blood loss amounted to 509.46 ± 302.61 mL. Furthermore, the ratio of intraoperative blood loss to estimated body volume was 16.16 ± 9.00%, and allogenic blood transfusions were administered in 3 out of 55 cases (5.45%).

The preoperative major Cobb angle was averaged at 58.24 ± 15.99°, and after corrective surgery, a substantial initial correction was observed, resulting in a postoperative major Cobb angle of 23.47 ± 8.02°. The major Cobb angle was maintained at 24.62 ± 7.72° at the last follow-up, indicating a long-term positive and sustained correction. The initial correction rate, calculated at 58.88 ± 12.88%, highlights the efficacy of the surgical intervention. Despite a slight loss in correction over time, as evidenced by the correction rate of 1.15 ± 3.36%, the last follow-up correction rate remained comparable at 58.88 ± 12.88%. Regarding the subgroup analysis between age groups (age 12–16 vs. age >16), the preoperative major Cobb angle for patients aged 12–16 years was 61.87 ± 17.51°, compared to 53.19 ± 12.25° for those aged >16 years, with a statistically significant difference indicated by a *p*-value of 0.046. Postoperatively, both age groups showed similar major Cobb angles, with values of 24.27 ± 8.80° and 22.36 ± 6.82°, and no significant difference (*p* = 0.390). At the last follow-up, the major Cobb angles were 25.20 ± 8.22° and 23.82 ± 7.07° for the respective age groups, with a non-significant *p*-value of 0.517. The initial correction rate did not differ significantly between age groups, recording values of 60.00 ± 13.35% for ages 12–16 and 57.16 ± 12.30% for ages >16 (*p* = 0.426). Similarly, the loss of correction rate and the last follow-up correction rate showed no significant age-related variations, with *p*-values of 0.675 and 0.348, respectively. These findings emphasize the comparable radiological results between different age groups receiving AIS corrective surgeries (Table 1). The 55 patients were satisfied with this deformity correction surgery for AIS.

**Figure 2:**
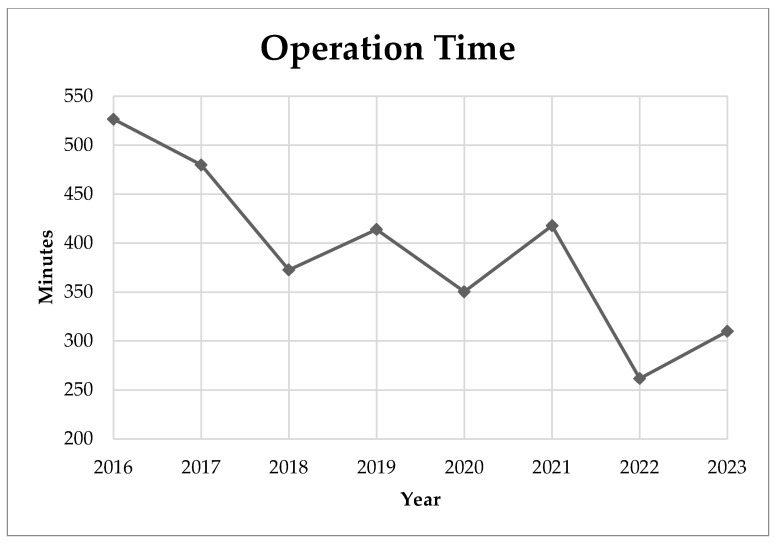
Mean operation time by year.

**Figure 3:**
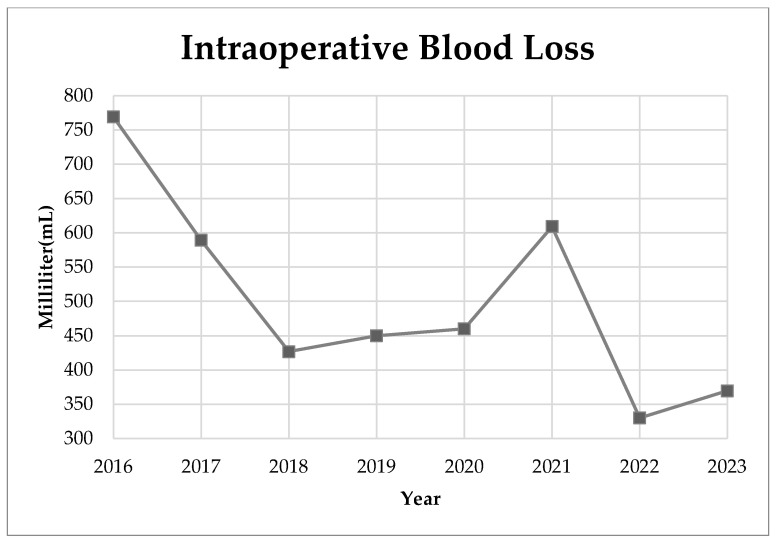
Mean intraoperative blood loss by year.

In our study, the perioperative complications associated with AIS surgery, as categorized by the Harms Study Group, were examined. The data are structured into two main categories: major and minor complications. In particular, there were no reported cases of death during the perioperative period of the examined surgeries. In terms of major complications, there were two cases accounting for 3.64% of the participants, while brachial plexus injury and need for revision surgery each occurred once, comprising 1.82% of the cases, respectively. In general, perioperative complications, both major and minor, had a rate of 3.64%**.** We recorded the operation time and intraoperative blood loss in AIS corrective surgeries from 2016 to 2023. During this period, the operation time showed a declining trend, reaching its lowest in 2022 at 411.67 min (Figure 2). Blood loss varied, with fluctuations throughout the years, but showed a general decrease from 2016 to 2023. The lowest recorded mean blood loss was 330 mL in 2022 (Figure 3). Between the two age groups (age 12–16 vs. age >16), both the operation time (*p* = 0.666) and intraoperative blood loss (*p* = 0.224) did not show significant difference.

## 4. Discussion

The reported overall postoperative major Cobb angle correction rate for AIS corrective surgery ranged from 58.7% to 71.4% [12,13,14]. Our study reports a substantial initial correction after surgery, highlighting the effectiveness of O-arm navigation AIS corrective surgery. The initial correction rate, calculated at 58.88 ± 12.88%, emphasizes the ability of spinal surgery to rectify a significant portion of the deformity. Despite a slight loss in correction over time, as indicated by the loss of correction rate of 1.15 ± 3.36%, the last follow-up correction rate remains comparable at 56.56 ± 13.27%. This suggests that the positive impact achieved immediately after surgery is primarily maintained over the follow-up period, consistent with previous studies that reported a 4.8% loss of correction rate during a ten-year follow-up and a mean loss of Cobb angle correction of 5.3 degree over an average twenty-year follow-up [32,33]. Meanwhile, reasearch has revealed an average 50.5 ± 23.1% of major Cobb angle correction rate after a minimum ten-year follow-up study. Furthermore, they achieved a postoperative Cobb angle correction rate of 64.0 ± 15.8% in patients with AIS [34]. Comparatively, we achieved a relatively small loss of correction rate; this could be attributed to the utilization of all pedicle screws under O-arm navigation that can precisely design the screw insertion track to increase the pull-out strength, avoiding implant loosening and acquiring a solid deformity correction. However, we also had a relatively short mean follow-up period of 932.7 days. Our results are consistent with previous studies, which achieved immediate and sustainable correction effects.

Surgeons vary widely in screw density during corrective surgeries for scoliosis, ranging from 1.27 to 1.92 implants per fused level, resulting in a mean correction of the major curve of 58.1% to 69.9% [14,19,35]. The growing utilization of pedicle screws has led to numerous studies on the impact of screw density. Some suggest slightly better radiographic correction with higher implant density [36,37], while others find no significant correlation between screw density and correction rate [38,39,40]. In our study, the mean screw density per fused level was 1.65 ± 0.25, which was considered moderate to high density. However, there is a lack of consensus on the cut-off point between low and high screw density [41,42]. Furthermore, there is a scarcity of reports focusing on the long-term outcome differences between high and low screw density for AIS patients. The average hospitalization duration in our study was 6.62 ± 1.16 days. Based on previous findings, the average duration of hospitalization for AIS surgery shows considerable variability, spanning from 3.5 to 13.5 days [14,43,44]. Extended length of hospitalization for scoliosis surgery is correlated with increased costs and a greater risk of postoperative complications within 90 days [45]. An adequate perioperative protocol to reduce the length of hospitalization for this surgery holds potential advantages for patients.

Subgroup analysis between age groups (age 12–16 vs. age >16) was performed, and none of the radiological surgery results were significant, except the Cobb angle of the preoperative period. Although Lonner et al. similarly reported no significant differences in the Cobb angle correction rate between adult and adolescent scoliosis surgery, adult scoliosis surgery had a relatively higher operative complication rate, which is, however, still considered low [46]. Another previous study reported that postoperative alignment was markedly poorer in patients with adult idiopathic scoliosis than in their adolescent counterparts [47]. Due to the possible rapid progression of AIS, early intervention including non-surgical and surgical intervention was recommended [48]. The purpose of scoliosis surgery is to halt the progression of the curve by achieving a robust fusion, correcting the deformity, and improving the cosmetic appearance. Adolescent corrective surgery, at an early or late stage, should be considered if indications are met. However, early intervention might achieve a more favorable outcome.

In this study, we achieved a mean intraoperative blood loss of 509.46 ± 302.61 mL and a mean surgery duration of 402.1 min with a mean total fused level of 11.55 levels. The operation time of our data is relatively longer than previous studies that focused on the posterior approach procedure, which reported 143.9 min with 10.3 fused levels [14], 255 min with 11 fused levels [43], and 351.5 min with 10.6 fused levels [49]. The longer operation time could result from routine setup for the intraoperative neuromonitoring device, positioning and padding, and the introduction of O-arm navigation, including preoperative and intraoperative settings. Furthermore, a study revealed that there is a lack of a standard definition of operative time for AIS surgery [50], which could cause difficulties in comparing the operation time data from different studies. However, we achieved considerably lower intraoperative blood loss compared to other studies, while the three previously mentioned studies reported mean intraoperative blood loss ranging from 945.4 to 1615 mL [14,43,49]. The navigation-assisted pedicle screw implantation technique improves the precision of the pedicle screw tract and prevents additional osteotomy which could play a crucial role in reducing intraoperative blood loss. In trend analysis, the mean operating time, although fluctuating by year, showed a general improvement from 526.6 min for year 2016 to 309.8 min for year 2023 (Figure 2). Similarly, we also achieved a general decrease in intraoperative blood loss from 768.9 mL for 2016 to 369.6 mL for 2023 (Figure 3). Both improvements can be credible indicators of our improvement and maturation in the AIS surgical technique.

**Figure 4:**
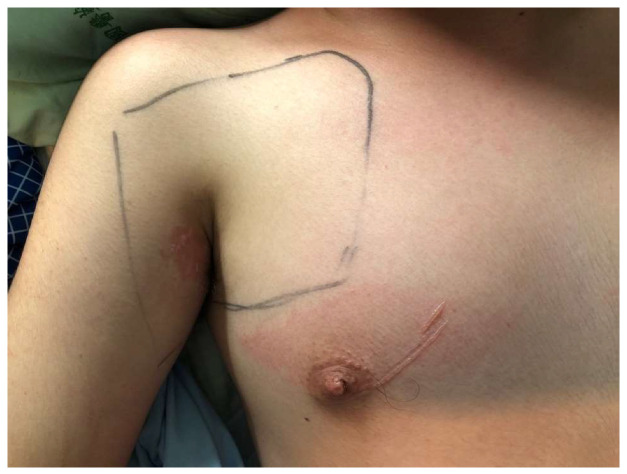
One case with postoperative brachial plexus injury due to inadequate padding.

**Figure 5:**
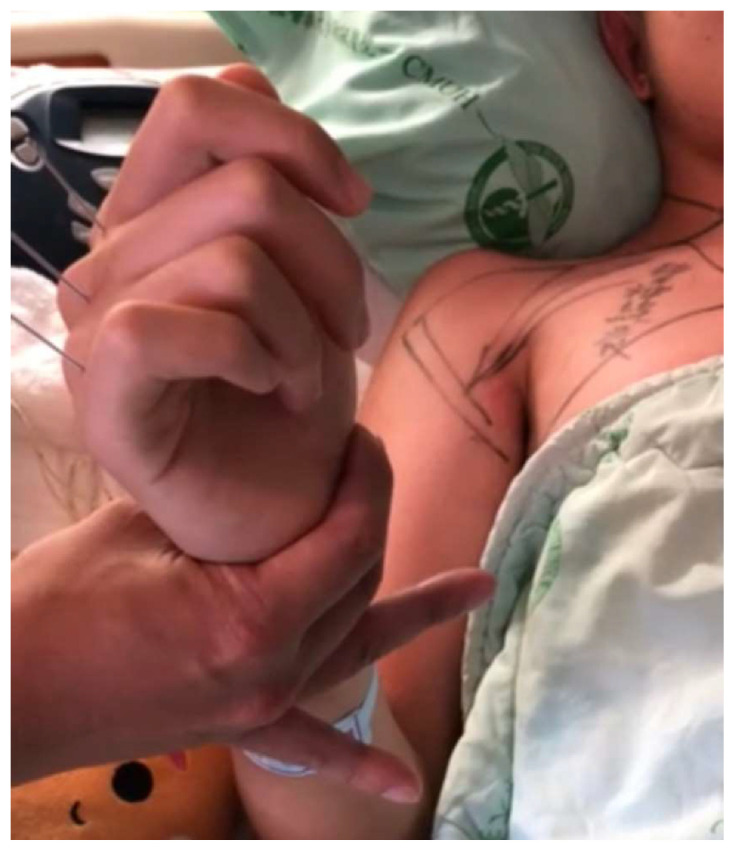
A patient with brachial plexus injury treated with acupuncture therapy and rehabilitation.

**Figure 6:**
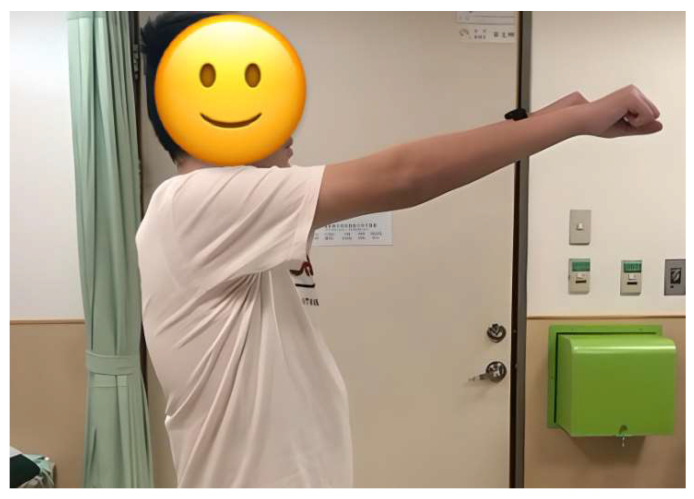
A patient with right brachial plexus injury recovered after acupuncture therapy and rehabilitation for 6 months.

**Figure 7:**
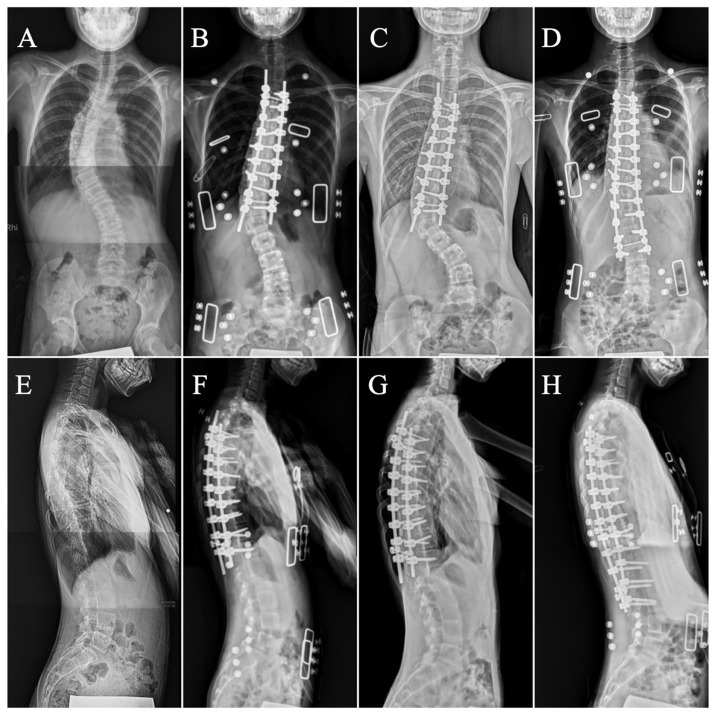
Postoperative decompensation of the lumbar curve and after revision surgery, (**A**) preoperative AP X-ray, (**B**) postoperative day 7, (**C**) postoperative decompensation year 3, (**D**) postrevision surgery day 7, (**E**) preoperative day 5 lateral X-ray, (**F**) postoperative day 7, (**G**) postoperative progression year 3, and (**H**) post-revision surgery day 7.

In our study, two cases experienced major complications, one with brachial plexus injury, and the other progressed to postoperative alignment decompensation that required revision surgery. Brachial plexus injury, mainly caused by malpositioning in surgery, was previously reported with an increased incidence in prone position spinal surgery [51]. Principles of prevention involve avoiding shoulder girdle depression, head rotation, shoulder extension, arm abduction beyond 70 degrees with elbow extension, external rotation beyond 60 degrees, neck extension, and abdominal compression [52]. For our patient, the patient was successfully treated with acupuncture and aggressive rehabilitation management and fully recovered after 6 months (Figure 4, Figure 5 and Figure 6). In general, revision surgeries for patients with AIS were indicated by residual rib deformity, implant failure, and progression of the unfused compensatory curve [53]. In this patient, revision surgery was required due to the rapid postoperative progression of the unfused compensatory lumbar curve. Initially, the patient received T4–T12 fusion with 18 screws implanted, and we extended the implantation to L3 with 6 additional pedicle screws during revision surgery (Figure 7). After revision surgery, we achieved ideal correction with good patient satisfaction. In our study, there were no implant-related complications, such as misplaced pedicle screws or loosening of the pedicle screw, etc. We believe that the O-arm navigation-assisted technique played a crucial role in enhancing the precision of screw instrumentation and lowering the implant-related complication rate.

Our study had several limitations, as it was specifically addressing perioperative radiological outcomes and complications for patients with AIS, lacking long-term results and further subjective measurements based on the patient. As a retrospective case series, we did not include a control group in our study to provide comparison to non-navigated surgeries. Due to inclusion of recent surgeries, some patients’ follow-up times were relatively short and may impact the loss of correction data. Due to our relatively small number of patients, we did not perform a subgroup analysis between Lenke types and between low and high screw density groups. The diversity in Lenke types and screw density among patients could influence surgical strategy and the selection of fusion levels, potentially introducing bias. Additional future research is necessary to explore these issues further.

## 5. Conclusions

Compared to the current literature, we believe that navigation plays an important role in facilitating pedicle screw instrumentation, reducing blood loss and implant-related complication, and lowering the loss of correction rate. This study indicated that patients receiving AIS surgery using the O-arm navigation-assisted one-stage posterior instrumentation method achieved significant and sustained clinical and radiological results.

## Figures and Tables

**Table 1:** Comparison of radiological surgery results between age groups.

Radiological Measurement	Total (n = 55)	Age 12–16 (n = 32)	Age > 16 (n = 23)	*p*-Value (Age) 12–16 vs. >16
Preoperative major Cobb angle (°)	58.24 ± 15.99	61.87 ± 17.51	53.19 ± 12.25	*p* = 0.046 *
Postoperative major Cobb angle (°)	23.47 ± 8.02	24.27 ± 8.80	22.36 ± 6.82	*p* = 0.390
Last follow-up major Cobb angle (°)	24.62 ± 7.72	25.20 ± 8.22	23.82 ± 7.07	*p* = 0.517
Initial correction rate (%)	58.88 ± 12.88	60.00 ± 13.35	57.16 ± 12.30	*p* = 0.426
Loss of correction rate (%) Last follow-up correction rate (%)	3.36 ± 11.09	2.82 ± 11.40	4.10 ± 10.87	*p* = 0.675
	56.56 ± 13.27	57.99 ± 13.93	54.56 ± 12.31	*p* = 0.348

Data are presented in terms of mean ± standard deviation values. * Indicated statistically significant difference between different time points, *p* value < 0.05.

## Data Availability

The data presented in this study are available on request from the corresponding author.
